# VM-CAGSeg: a vessel structure-aware state space model for coronary artery segmentation in angiography images

**DOI:** 10.3389/fmed.2025.1661680

**Published:** 2025-10-10

**Authors:** Yuanqing He, Zhenhuan Lyu, Yayue Mai, Si Li, Chen-kai Hu

**Affiliations:** ^1^School of Artificial Intelligence and Digital Economy Industry, Guangzhou Institute of Science and Technology, Guangzhou, China; ^2^Department of Cardiology, Second Affiliated Hospital of Nanchang University, Nanchang, China

**Keywords:** vessel structure-aware state space model, coronary angiography, vessel segmentation, cross-stage feature interaction fusion, Kolmogorov-Arnold state space, Frangi filter

## Abstract

Coronary artery segmentation in X-ray angiography is clinically critical for percutaneous coronary intervention (PCI), as it offers essential morphological guidance for stent deployment, stenosis assessment, and hemodynamic optimization. Nevertheless, inherent angiographic limitations, including complex vasculature, low contrast, and fuzzy boundaries, persist as significant challenges. Current methodologies exhibit notable shortcomings, including fragmented output continuity, noise susceptibility, and computational inefficiency. This study proposes VM-CAGSeg, a novel U-shaped architecture integrating vessel structure-aware state space modeling, to address these limitations. The framework introduces three key innovations: (1) A Vessel Structure-Aware State Space (VSASS) block that synergizes geometric priors from a Multiscale Vessel Structure-Aware (MVSA) module with long-range contextual modeling via Kolmogorov–Arnold State Space (KASS) blocks. The MVSA module enhances tubular feature representation through Hessian eigenvalue-derived vesselness measures. (2) A Cross-Stage Feature Interaction Fusion (CSFIF) module that replaces conventional skip connections with cross-stage feature fusion strategies to enhance the variability of learned features, preserving long-range dependencies and fine-grained details. (3) A unified architecture that integrates the Vessel Structure-Aware State Space (VSASS) block and the Cross-Stage Feature Interaction Fusion (CSFIF) module to achieve comprehensive vessel segmentation by synergizing multiscale geometric awareness, long-range dependency modeling, and cross-stage feature refinement. Experiments demonstrate that VM-CAGSeg achieves state-of-the-art performance, surpassing CNN-based (e.g., UNet++), transformer-based (e.g., MISSFormer), and state space model (SSM)-based (e.g., H_vmunet) methods, with a Dice similarity coefficient (DSC) of 88.15%, mIoU of 79.19%, and a 95% Hausdorff distance (HD95) of 13.68 mm. The framework significantly improved boundary delineation, reducing HD95 by 49.8% compared to UNet++ (27.15 mm) and by 16.6% compared to TransUNet (15.85 mm). While its sensitivity (90.05%) was marginally lower than that of TransUNet (90.33%), the model's balanced performance in segmentation accuracy and edge precision confirmed its robustness. These findings validate the effectiveness of integrating multiscale vessel-aware modeling, long-range dependency learning, and cross-stage feature fusion, making VM-CAGSeg a reliable solution for clinical vascular segmentation tasks that require fine-grained detail preservation. The proposed method is available as an open-source project at https://github.com/GIT-HYQ/VM-CAGSeg.

## 1 Introduction

Vessel segmentation in coronary angiography can provide valuable clinical information for percutaneous coronary intervention (PCI). Invasive X-ray coronary angiography plays a crucial role in the diagnosis and treatment of coronary heart disease (1). Vascular segmentation can extract coronary artery morphology from complex angiographic images, eliminate the interference of surrounding tissues, and clearly display the location, length, and morphological characteristics of vascular stenosis, calcifications, and bifurcation lesions (2). In preoperative planning, vascular segmentation can help cardiologists predict the path of the guidewire, select the length and diameter of the stent, and determine the strategy for balloon expansion. Intraoperative navigation and vascular segmentation combined with real-time imaging can assist in adjusting the catheter angle to reduce the difficulty of instrument passage due to vascular distortion. The results of vessel segmentation can be combined with flow reserve fraction (FFR) or quantitative coronary angiography (QCA) to quantify the effect of stenosis on blood flow (3, 4). After operation, the lumen diameter and blood flow velocity before and after stenosis are divided and compared to verify the effect of stent adhesion and expansion. In complex scenarios, such as bifurcation lesions, vascular segmentation can fuse multi-angle angiographic data, reconstruct three-dimensional vascular paths, and identify microchannels or collateral circulation. A three-dimensional vascular model combined with intravascular ultrasound (IVUS) and optical coherence tomography (OCT) enables multi-modal image fusion and improves the pre-treatment accuracy of calcification lesions (5). Clinical applications demand anatomically precise vessel delineation that is robust to pathological alterations across diverse vascular structures and imaging modalities. Surgical navigation requires temporally stable segmentation enabling real-time instrument tracking and seamless integration with intraoperative imaging workflows.

**Figure 1:**
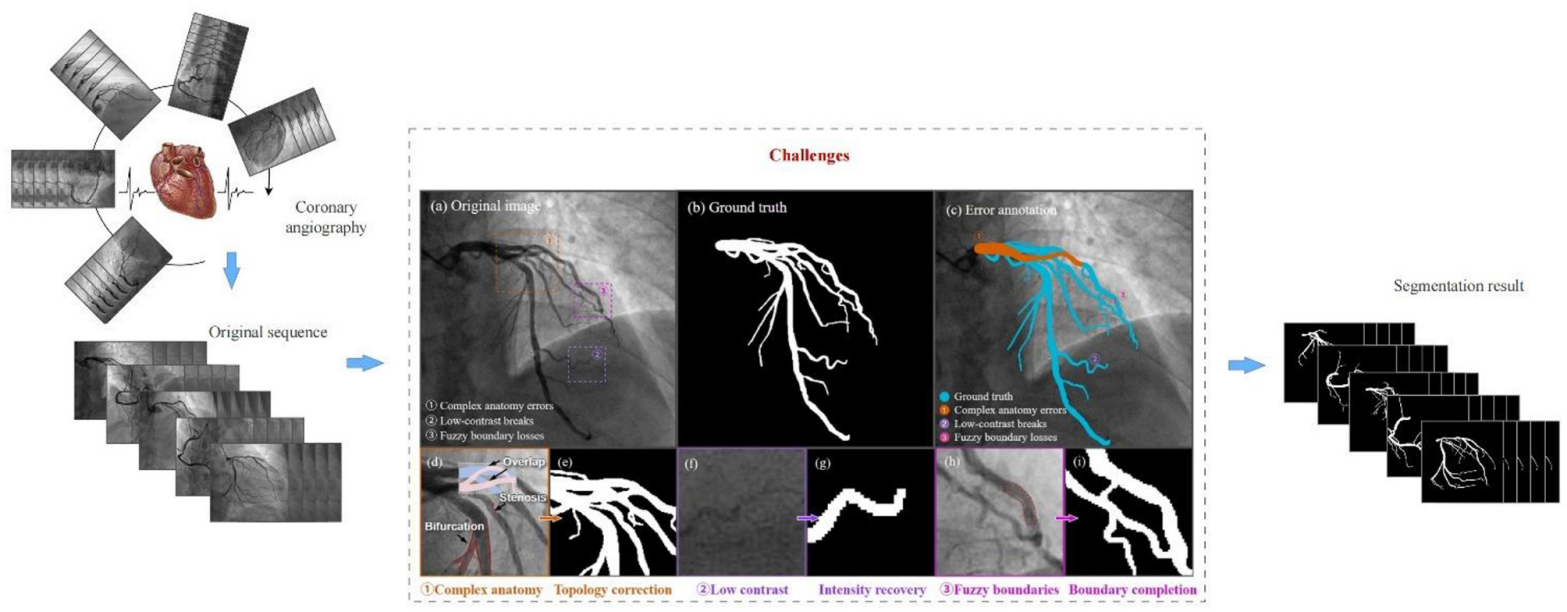
Brief illustration of challenges.

Complex vascular structures, low contrast, and fuzzy vascular boundaries are the key challenges in coronary segmentation, as shown in Figure 1. First, the coronary artery system exhibits highly complex morphological characteristics, extending beyond simple tree-like branching. Coronary arteries branch hierarchically from main trunks into finer vessels, with significant inter-patient variations in bifurcation patterns. In 2D angiographic projections, vessels frequently appear crossed or intertwined due to overlapping perspectives. Cardiac motion and respiration induce non-rigid deformation of vascular structures across temporal sequences (6). Second, low contrast refers to the weak grayscale difference between the coronary arteries and the surrounding tissues. This arises from non-uniform contrast agent distribution, background noise interference, and tissue overlap artifacts. Variations in blood flow velocity lead to insufficient contrast filling in small vessels or stenotic lesions, resulting in localized low-intensity signals or apparent vascular discontinuity. High-frequency noise from bones or catheters in X-ray imaging can mimic vascular textures (especially in low-dose angiography), causing conventional threshold-based segmentation to misidentify noise as vessels. In 2D angiography, overlapping anatomical structures (e.g., myocardium, valves) generate pseudo-vessel signals (e.g., right coronary artery overlapping with spinal shadows) (7). Finally, unclear vascular boundaries further complicate segmentation. This problem results from motion artifacts, partial volume effects, and pathological interference. Rapid cardiac systolic motion causes edge smearing, especially in low-frame-rate angiography systems. Limited imaging resolution blends boundary pixels of small vessels with adjacent tissues, creating semi-transparent blurred edges. Local high-intensity signals from calcified plaques or stent metal artifacts obscure true vessel walls, while atherosclerotic plaques may cause irregular lumen boundaries (8).

Addressing coronary segmentation problems is still challenging. Recent advances in neural network-based coronary artery segmentation in angiography images can be divided into three categories: CNN-based approaches, transformer-based approaches, and state space model (SSM)-based approaches. The first category encompasses CNN-based architectures, primarily U-Net variants and attention-enhanced models. As a pioneering end-to-end segmentation framework, U-Net (9, 10) has demonstrated remarkable performance in medical image analysis. Owing to its simple yet effective structure, high scalability, and proven segmentation efficacy, numerous subsequent works have extended this U-shaped architecture through various enhancements. For example, UNet++ (11) introduces dense skip connections to replace the original simple connections, thereby strengthening feature representation and mitigating information loss during downsampling. Similarly, Attention U-Net (12) incorporates attention gates to dynamically weight feature importance, enabling the model to focus adaptively on target regions. While understanding the global context is essential for medical image segmentation, CNN-based architectures are fundamentally constrained by their local receptive fields, limiting their ability to capture long-range dependencies. The second category consists of transformer-based architectures. Transformer-based architectures have gained significant traction in medical image segmentation following the success of the vision transformer (13) in general computer vision tasks. These models address the inherent limitation of CNN-based architectures in capturing long-range dependencies by leveraging the global receptive field of self-attention mechanisms. For instance, TransUNet (14) pioneered the integration of transformers in medical segmentation, combining a CNN encoder with a transformer-based decoder to effectively capture both local and global features. TransFuse (15) further advanced this paradigm by using a parallel hybrid encoder, where CNN and ViT branches process local and global features separately before fusion. Swin-UNet (16) introduced the first pure transformer-based U-Net, utilizing hierarchical Swin Transformer (17) blocks for efficient multi-scale representation learning. To enhance feature diversity, DS-TransUNet (18) processes multi-scale image patches through dual parallel Swin Transformer encoders. Meanwhile, OCT^2^Former (19) proposes a hierarchical hybrid transformer to refine boundary details, while MISSFormer (20) optimizes long-range modeling with cross-scale self-attention for improved organ segmentation. Despite their superior modeling of global relationships, transformer-based approaches suffer from quadratic computational complexity relative to input size, imposing significant memory and processing demands. The third category, SSM-based architectures, offers a promising alternative to CNNs and transformers for medical image segmentation. Mamba's linear-time sequence modeling capability and input-dependent state transition mechanism address the computational limitations of transformers while maintaining global receptive fields. Subsequent improvements led to VMamba (21), which introduced cross-scanning mechanisms to better capture 2D spatial relationships in medical images. Several medical segmentation approaches have adapted these SSM-based designs. VM-UNet (22) pioneered the integration of Mamba blocks into U-Net architectures, demonstrating efficient long-range modeling for medical image segmentation. H_vmunet (23) enhanced hierarchical feature learning through high-order spatial interactions, achieving superior vessel segmentation. SegMamba (24), a hybrid SSM-CNN model, has begun to demonstrate the potential of SSM-based models in medical image segmentation.

Current SSM-based vessel segmentation frameworks in coronary angiography have critical limitations. First, inadequate geometric modeling of tubular structures fails to leverage inherent vascular morphological priors, compromising boundary delineation in high-curvature or low-contrast regions. Second, limited topological modeling capability causes errors at bifurcations, crossings, and distal branches, disrupting vascular connectivity. Finally, ineffective management of angiographic noise and dynamic variations yields temporally inconsistent segmentation with poor signal-to-noise (SNR) robustness. These shortcomings demand the development of novel architectures that specifically address the geometric, topological, and dynamic characteristics of coronary vessels.

In this study, we propose VM-CAGSeg, a Vessel Structure-Aware State Space Model for coronary artery segmentation, that integrates Visual State Space Models (VSSMs) (21) within a U-Net architecture. The framework addressed three critical limitations through the following approaches: (1) A Vessel Structure-Aware State Space (VSASS) block synergizing geometric priors with efficient long-range modeling, (2) a Multiscale Vessel Structure-Aware (MVSA) component using Frangi filtering (25) to explicitly represent tubular structures across scales, (3) Kolmogorov–Arnold State Space (KASS) blocks enabling linear-complexity global context integration, and (4) a Cross-Stage Feature Interaction Fusion (CSFIF) module replacing skip connections to preserve hierarchical spatial-semantic features.

The main contributions to this study are as follows:

1) A novel VSASS block that integrates an MVSA component with KASS modules, jointly capturing tubular morphology and long-range dependencies to overcome limitations in current SSM-based medical segmentation.
2) A CSFIF module that replaces conventional skip connections with cross-stage feature fusion strategies, enhancing feature diversity while preserving long-range contextual relationships and fine-grained vascular details.
3) A unified architecture synergizing VSASS blocks and CSFIF modules, achieving comprehensive vessel segmentation through simultaneous multiscale structural awareness, global dependency modeling, and cross-stage feature refinement.

## 2 Related work

### 2.1 Frangi vessel enhancement filter

The Frangi vessel enhancement filter (25) is based on the eigenvalue analysis of the Hessian matrix on multiple Gaussian scales. Given a location *x* in the image domain Ω, the vascular response function is directly related to the characteristic values of the Hessian matrix at that location. The Hessian matrix consists of the second derivative of the image intensity at *x*, defined by the following (Formula 1):


(1) $$H(x)=\left[\begin{array}{cc} I_{xx}(x) & I_{xy}(x)\\ I_{yx}(x) & I_{yy}(x) \end{array}\right]\;$$

The noisy image is generally first de-noised by Gaussian filters, defined by Formula 2:


(2) $$\begin{array}{l} G\left(x;y,\sigma \right)=\frac{1}{2\pi \sigma^{2}}e^{-\frac{||y-x|{|}^{2}}{2\sigma^{2}}}\; \end{array}$$

The two eigenvalues of the Hessian matrix are denoted by λ_1_ and λ_2_ ($\left|\begin{array}{c} \lambda_{2} \end{array}|\geq |\begin{array}{c} \lambda_{1} \end{array}\right|$), which are calculated using the following (Formula 3):


(3) $$\begin{array}{l} \lambda_{1,2}=\frac{\left(I_{xx}+I_{yy}\right)\pm \sqrt{{\left(I_{xx}-I_{yy}\right)}^{2}+4I_{xy}^{2}}}{2}\; \end{array}$$

Vascular structures are obtained if λ_1_ and λ_2_ satisfy the following conditions: ||λ_1_||≈0 and ||λ_2_||≫||λ_1_||. The response function of the vascular structures that are darker than the background in a 2D image is defined using the following (Formula 4):


(4) $$V_{0}(s)=\{\begin{array}{c} 0,\;if\;\lambda_{2}>0\\ exp(-\frac{R_{B}^{2}}{2\beta^{2}})(1-\exp(-\frac{S^{2}}{2c^{2}})) \end{array}$$

where $s=\sqrt{\lambda_{1}^{2}+\lambda_{2}^{2}}$ is the second-order structureness. $R_{B}=\frac{\left||\lambda_{1}|\right|}{\left||\lambda_{2}|\right|}$ is the blobness measure in 2D and accounts for the eccentricity of the second-order ellipse. β and *c* are thresholds that control the sensitivity of the line filter to the blobness and structureness terms. β is fixed to 0.5. The value of the threshold c depends on the greyscale range of the image, and half the value of the maximum Hessian norm has proven to work in most cases. In a previous study (26), the Frangi filter provided optimal vesselness enhancement for multiscale region growing (MSRG), enabling 80% coronary tree coverage by fusing Hessian-based features with directional data to preserve continuity. In another study (27), an enhanced Frangi filter extracted Hessian-derived edges from noisy angiograms, synergizing with optical flow-based motion blur to reconstruct the full arterial topology across 50 patient videos. In previous research (28), as a core component in a multi-filter ensemble (Frangi/modified Frangi/MFAT), the Frangi filter contributed complementary features for weighted-median fusion, boosting CTA segmentation beyond individual filters. In a separate study (29), Frangi-based multiscale enhancement optimized vessel-background contrast for level-set segmentation, validated on retinal vessels, with coronary transferability discussed.

### 2.2 Kolmogorov–Arnold networks (KANs)

Kolmogorov–Arnold networks (KANs) (30) are neural networks based on the Kolmogorov–Arnold representation theorem, which provides a theoretical foundation for approximating arbitrary multivariate continuous functions. Originally formulated by Andrey Kolmogorov and Vladimir Arnold, the theorem states that any n-dimensional continuous function can be expressed as a finite composition of univariate functions, as shown in the following (Formula 5):


(5) $$\begin{array}{l} f\left(x_{1},x_{2},...,x_{n}\right)=\overset{2n+1}{\underset{i=1}{\sum }}\varphi_{i}\left(\overset{n}{\underset{j=1}{\sum }}\psi_{ij}\left(x_{j}\right)\right)\; \end{array}$$

where φ_*i*_ and ψ_*ij*_ are continuous univariate functions. This decomposition implies that complex multivariate functions can be approximated through linear combinations and non-linear transformations of simpler one-dimensional functions.

Inspired by the Kolmogorov–Arnold representation theorem, Kolmogorov–Arnold networks (KANs) use a three-layer neural network architecture to approximate complex functions in high-dimensional input spaces, which comprises five key components: an input layer, a mapping layer, a combination layer, a non-linear activation layer, and an output layer. The complete operations of Kolmogorov–Arnold networks (KANs) are formally defined as follows (Formula 6):


(6) $$\begin{array}{l} \begin{array}{c} X=\left[x_{1},x_{2},...,x_{n}\right]\\ z_{ij}=\psi_{ij}\left(x_{i}\right)\\ h_{i}=\sum_{j=1}^{n}z_{ij}\\ f\left(x\right)=\sum_{i=1}^{2n+1}\varphi_{i}\left(h_{i}\right) \end{array}\; \end{array}$$

where is the multidimensional vector of input, *z*_*ij*_ represents the one-dimensional features after mapping, ψ_*ij*_ is the mapping function, *h*_*i*_ is the new feature representation obtained by linearly combining the outputs of the mapping layer, φ_*i*_(*h*_*i*_) is the non-linear activation function applied to the output of the combination layer, and *f*(*x*) is the final output after superposition of all non-linearly activated features.

To improve the computational efficiency of KANs, Li proposed FastKAN (31), which uses Gaussian radial basis functions (RBFs) to approximate the B-spline basis, a major bottleneck in KANs. This is defined by the following (Formula 7):


(7) $$\begin{array}{l} \varphi \left(h\right)=\exp\left(-\frac{h^{2}}{2p^{2}}\right) \end{array}$$

where *h* is the radial distance and *p* is the parameter that controls the width or spread of the function.

To further improve computational efficiency, Athanasios Delis proposed FasterKAN (32), which uses the Reflectional Switch Activation Function (RSWAF) to approximate the B-spline basis. This is 1.5 × faster than FastKAN, as shown in the following (Formula 8):


(8) $$\begin{array}{l} \varphi \left(h\right)=1-{\left(\tanh\left(\frac{h}{p}\right)\right)}^{2} \end{array}$$

where *h* is the radial distance and *p* is the parameter that controls the width or spread of the function. In a previous study (33), KAN-based modules (KAN-ACM/KAN-BM) in KANSeg resolved blurred organ boundaries, achieving a 90.99% Dice score on cardiac data via non-linear feature learning. In another study (34), MM-UKAN++ used multilevel KAN layers with attention mechanisms for ultrasound segmentation, attaining a Dice score of 81.30% at 3.17 G FLOPS, outperforming CNNs and transformers. In a separate study (35), KAN-MambaNet integrated learnable activation functions to distinguish myocardial edema/scar boundaries, overcoming small-region limitations in cardiac MRI. In previous research (36), proKAN's B-spline-based KAN blocks prevented overfitting in liver tumor segmentation while maintaining interpretability, reducing computational overhead through progressive stacking.

### 2.3 Deep learning-based coronary angiogram segmentation

These studies proposed advanced deep learning architectures to address coronary angiography segmentation challenges. Hamdi et al. (37) introduced a GAN framework featuring a novel U-Net generator with dual self-attention blocks and an auxiliary path to enhance feature generalization for thin vessels. Bao et al. (38) developed SARC-UNet, which incorporates residual convolution fusion modules (RCFMs), for multi-scale feature integration and a location-enhanced spatial attention (LESA) mechanism to preserve vascular connectivity. Abedin et al. (39) incorporated Self-Organizing Neural Networks (Self-ONNs) into a U-Net architecture, utilizing DenseNet121 encoders and enhanced decoders for robust feature extraction, with additional integration into multi-scale attention networks for stenosis localization.

Entropy-aware gated (EAG)-scale-adaptive enhancement (SAE)-elastic spatial topology fusion (ESTF) (40) is a novel network for coronary artery segmentation in X-ray angiography. It integrates an entropy-aware gated module to suppress catheter interference, a scale-adaptive enhancement mechanism for multi-scale vessel extraction, and an elastic spatial topology fusion module to maintain vascular continuity. The architecture effectively addresses semantic confusion and topological fragmentation in complex coronary structures. While the “entropy-aware gated (EAG) module” is specifically designed to suppress semantic interference from catheters, the Frangi filter within our proposed MVSA module inherently enhances vascular structures while implicitly suppressing non-vascular interference, achieving similar catheter suppression effects through morphological prioritization. The EAG-SAE-ESTF framework proposed an SAE module to improve the structural representation of multi-scale coronary arteries. Similarly, the MVSA module in our study also used a multi-scale vessel enhancement architecture for analogous purposes. The EAG-SAE-ESTF method incorporates an ESTF module to perceive and model global information and directional topological structures. Our proposed approach integrated KASS blocks, enabling linear-complexity global context integration and a CSFIF module replacing skip connections to preserve hierarchical spatial-semantic features. Through these two modules, our method also effectively achieved the perception and modeling of vascular spatial topology.

## 3 Materials and methods

### 3.1 Overall architecture of VM-CAGSeg

**Figure 2:**
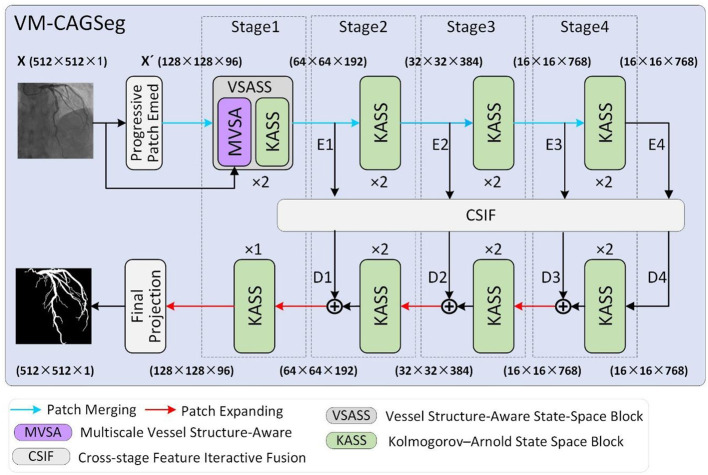
Overall illustration of the proposed VM-CAGSeg network.

Figure 2 illustrates the overall architecture of VM-CAGSeg. The proposed model consisted of four core components: an encoder, a decoder, a bottleneck, and skip connections, collectively forming a U-shaped architecture. A progressive patch embedding layer first partitioned the input grayscale image *X*∈ℝ^*H*×*W*×1^ into non-overlapping 4 × 4 patches. To preserve fine-grained details while enhancing non-linear representational capacity, this layer progressively mapped the spatial dimensions to a channel depth C (default to 96), producing an embedded image $X^{\prime }\in {\mathbb{R}}^{\frac{H}{4}\times \frac{W}{4}\times C}$. Subsequently, *X*′ underwent layer normalization (41) prior to encoder processing.

The encoder comprised four hierarchical stages. Stage 1 incorporated two of the proposed VSASS blocks, each integrating one proposed MVSA block and one proposed KASS block. Stages 2–4 each used two KASS blocks. A patch merging operation followed the first three stages, progressively halves the spatial dimensions while doubling channel depth according to the sequence [C, 2C, 4C, 8C].

The decoder mirrored this four-stage architecture. Stages 2–4 began with patch expanding operations that halved channel depth while doubling the spatial resolution. Each stage integrated [2, 2, 2, 1] KASS blocks respectively, with channel dimensions progressively decreasing according to the sequence [8C, 4C, 2C, C]. A final projection layer then upsampled features 4 × via patch expanding to recover the original spatial dimensions, followed by convolutional mapping to the segmentation space.

Inspired by CSPNet (42), we implemented cross-stage feature interaction using the proposed Cross-Stage Feature Interactive Fusion (CSIF) module within both bottleneck and skip connections. The CSIF module first fused adjacent stage features via convolutional operations, then performed element-wise summation with the corresponding decoder inputs during feature reconstruction.

### 3.2 Vessel structure-aware state space (VSASS) block

**Figure 3:**
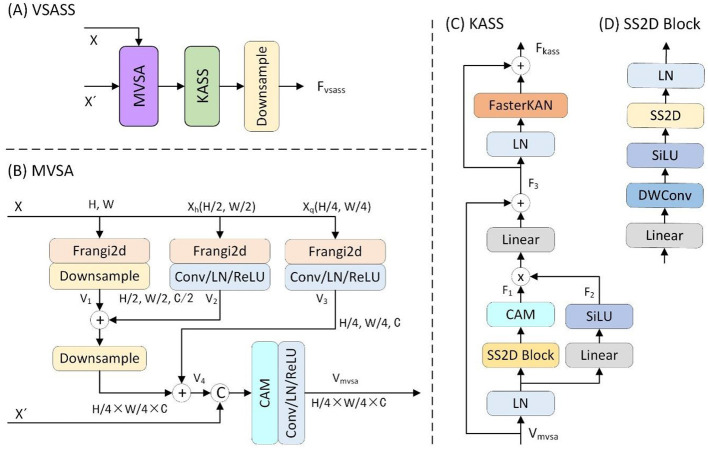
Details of the proposed **(A)** VSASS block and its sub-block **(B)** MVSA, **(C)** KASS and **(D)** SS2D block.

As detailed in Figure 3A, the proposed VSASS block integrated one proposed MVSA block and one proposed KASS block. Concurrently, Figure 2 illustrates the architectural workflow. *X*∈ℝ^*H*×*W*×1^ represents the original grayscale input image, while $X^{\prime }\in {\mathbb{R}}^{\frac{H}{4}\times \frac{W}{4}\times C}$ denotes the embedded image obtained through the progressive patch embedding module.

#### 3.2.1 Multiscale vessel structure-aware (MVSA) block

To reinforce the extraction of vascular structural features, we proposed the MVSA block. As detailed in Figure 3B, the proposed MVSA block first extracted vascular features at three spatial scales (1 × , 0.5 ×, 0.25 ×) from the input image *X*∈ℝ^*H*×*W*×1^ using a Frangi filter (25). The original scale feature map was then downsampled by a factor of two. The output *V*_1_ was added to the half-scale vascular features (*V*_2_). This combined output underwent further 2 × downsampling before summation with the quarter-scale features (*V*_3_). A channel attention module then processed these concatenated multi-scale features (*V*_4_) alongside the embedded features $X^{\prime }\in {\mathbb{R}}^{\frac{H}{4}\times \frac{W}{4}\times C}$, yielding the vascular-enhanced output *V*_*mvsa*_. This process is formally expressed in the following (Formula 9):


(9) $$\begin{array}{l} \;X_{h}=Downsampler\times 2\left(X\right)\\ \;X_{q}=Downsampler\times 4(X)\\ \;V_{1}=Downsampler\times 2(Frangi2D\left(X\right))\\ V_{2}=ReLU(LN(Conv((Frangi2D(X_{h})))))\\ V_{3}=ReLU(LN(Conv((Frangi2D(X_{q})))))\\ \;V_{4}=Downsampler\times 2(V_{1}+V_{2})+V_{3}\\ V_{mvsa}=ReLU(LN(Conv(CAM([V_{4},X^{\prime }])))) \end{array}$$

*X*_*h*_ and *X*_*q*_ are the 2 × and 4 × downsampled versions of the input *X*, respectively. *V*_1_, *V*_2_, and *V*_3_ denote the three-scale vascular features extracted using Frangi filtering, while *V*_4_ represents their fused features.

#### 3.2.2 Kolmogorov–Arnold state space (KASS) block

To enhance the model's capability for non-linear representation learning and complex topological modeling, we proposed the KASS block. As shown in Figure 3C, the proposed KASS block adapted VMamba's VSS block (14) with two key modifications: First, it replaced the original Feed-Forward Network (FFN) with a FasterKAN block (32), and second, it retained the multiplicative branch integrated with local attention for enhanced feature representation.

In the first branch, *X* passed sequentially through a linear layer, depthwise convolution, SiLU activation, and a 2D selective scan (SS2D) module for global feature extraction, followed by linear normaliztion (Linear) to produce the output *F*_1_. In the second branch, *X* passed through a linear layer, SiLU activation, and a local attention module, generating the output *F*_2_. The outputs of both branches were then element-wise multiplied, processed in another linear layer, and combined with a residual connection to produce the intermediate output *F*_3_.

In the second component, the feature *F*_3_ passed through layer normalization (LN) and a FasterKAN block (32). The result was combined with *F*_3_ via an additive residual connection, yielding the KASS output *F*_*kass*_. Finally, *F*_*kass*_ underwent 2 × spatial downsampling with concurrent 2 × channel expansion to produce the VSASS block output *F*_*vsass*_.

The overall computation is defined by the following (Formula 10):


(10) $$\begin{array}{c} F_{1}=CAM(LN(SS2D(SiLU(DWConv(Linear(LN(X))))))\\ F_{2}=SiLU(Linear(LN\left(V_{mvsa}\right)))\\ F_{3}=V_{mvsa}+Linear(F_{1}⊙F_{2})\\ F_{kass}=FasterKAN\left(LN\left(F_{3}\right)\right)+F_{3}\#\\ F_{vsass}=ChannelExpand\times 2(Downsampler\times 2\left(F_{kass}\right)) \end{array}$$

### 3.3 Cross-stage feature interactive fusion (CSIF) block

Standard skip connections directly concatenate shallow encoder features with deep decoder features. The significant semantic disparity between these features hinders effective fusion optimization. This limitation is particularly problematic for medical images with low-contrast boundaries. Furthermore, skip connections only aggregate features at identical scales, failing to leverage multi-scale contextual information. Consequently, segmentation performance is compromised for small targets and complex structures. To address these limitations, we introduced the Cross-stage feature interactive fusion (CSIF) module.

**Figure 4:**
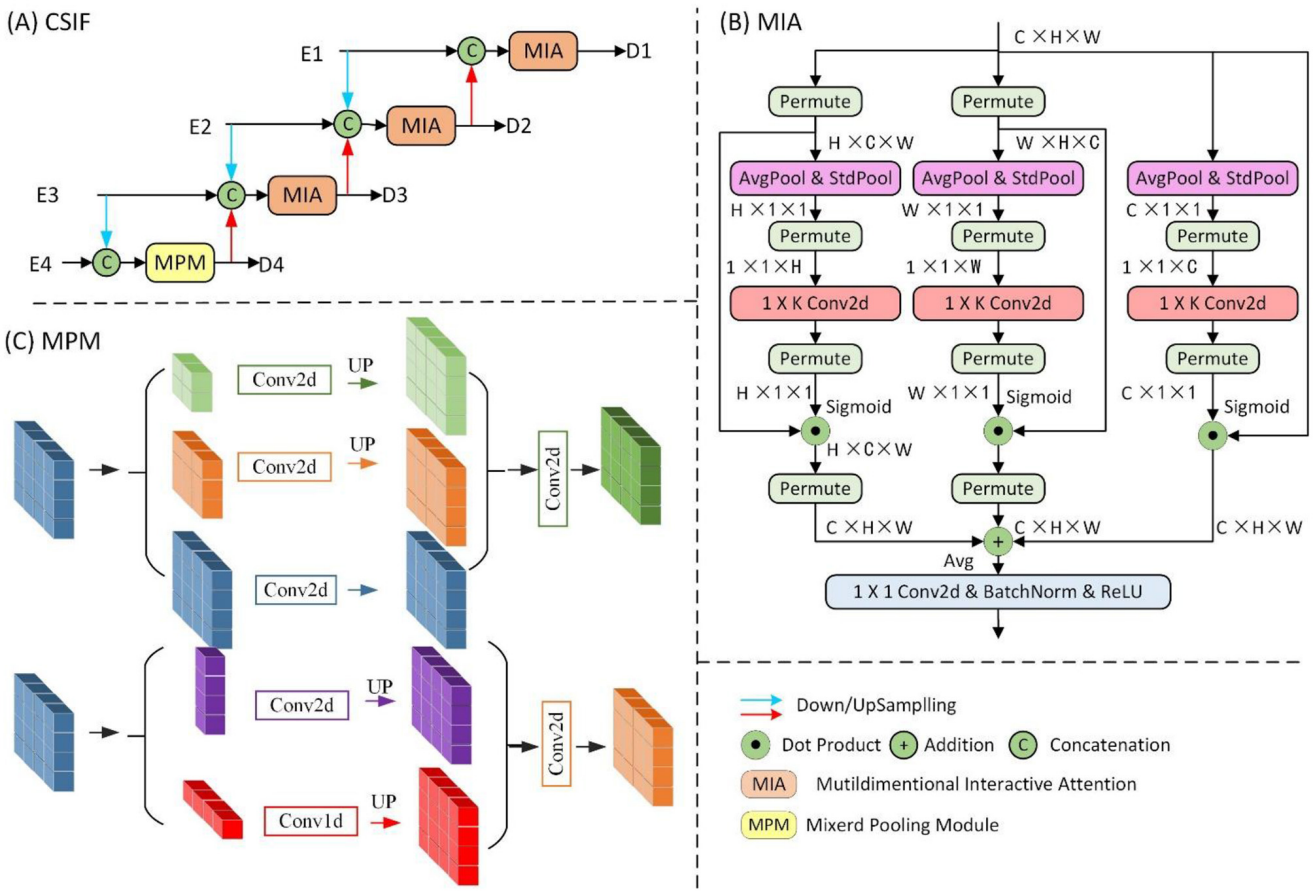
Details of the proposed **(A)** CSIF block and its sub-block **(B)** MPM and **(C)** MIA block.

The proposed CSIF module (Figure 4), inspired by the EFM module in CSPNet (42), adopts cross-stage feature fusion strategies, enhancing feature diversity across network layers through truncated gradient flow. The CSIF module processes four hierarchical encoder features [E1, E2, E3, E4] through a top-down refinement cascade to generate interactively fused outputs [D1, D2, D3, D4].

First, D4 is generated by fusing E3 and E4 through the Mixed Pooling Module (MPM) (43). Next, D3 integrates 2 × downsampled E2 with native-resolution E3 and prior-stage D4. Subsequently, D2 combines 2 × downsampled E1, 2 × upsampled D3, and current-stage E2. Finally, D1 fuses 2 × upsampled D2 with the encoder input E1. Critically, all fusion operations use Multiscale Interaction Aggregation (MIA) to enhance cross-stage feature compatibility.

The Mixed Pooling Module (MPM), as shown in Figure 4C, captures both short-range and long-range dependencies through parallel pathways: short-range modeling uses lightweight pyramid pooling plus convolutional layers for local context extraction, while long-range modeling leverages horizontal/vertical strip pooling to connect distant regions.

The MIA module, as shown in Figure 4B, uses a Multidimensional Collaborative Attention (MCA) (44) mechanism to extract attention-enhanced features. The three-branch architecture captures feature interactions across width (W), height (H), and channel dimensions through permute-based long-range dependency modeling, with final outputs integrated via averaging. Finally, the features are processed by convolutional layers, batch normalization (BN), and ReLU activation for non-linear transformation.

## 4 Experimental data and evaluation methods

### 4.1 Experimental data and parameters

#### 4.1.1 Clinical data

This study utilized a clinical dataset of 1,856 anonymized coronary angiography sequences retrospectively collected from 102 patients (38 males, 64 females; aged 45–82 years) at the Second Affiliated Hospital of Nanchang University (January 2022–December 2024). The cohort encompassed diverse cardiovascular pathologies: stable angina (37.2%), acute coronary syndrome (41.5%), and chronic total occlusion (21.3%). All angiograms were acquired using Phillips Xper FD10 systems at 15 frames/second, with resolutions ranging from 512 × 512 to 1,024 × 1,024 pixels, across standard projections (e.g., LAO 45, RAO 45, and AP views).

Ethical approval was granted by the Institutional Review Boards of the participating institutions, with informed consent waived for this retrospective study. The exclusion criteria eliminated frames with excessive motion artifacts, poor contrast filling, or prior coronary bypass grafts. A team of one interventional cardiologist and two medical imaging experts independently annotated all major coronary arteries (LAD, LCX, and RCA) using ITK-Snap (45).

The dataset was partitioned at the patient level into training (61 patients; 1,112 sequences), validation (21 patients; 371 sequences), and test sets (20 patients; 373 sequences). All images underwent standardized preprocessing with isotropic resampling to 512 × 512 pixels and intensity normalization. Data augmentation involved spatial transformations such as rotation and random flipping.

#### 4.1.2 Experimental parameters

The network was implemented in PyTorch 1.13 with CUDA 11.6 acceleration. Training used a batch size of 4 and the AdamW optimizer (42) with an initial learning rate *of* 1 × 10^−3^, β_1_ = 0.9, β_2_ = 0.999, ϵ = 1 × 10^−8^, weight decay λ = 1 × 10^−2^, and AMSGrad disabled. CosineAnnealingLR (43) was utilized as the scheduler with a maximum of 50 iterations and a minimum learning rate of 1 × 10^−5^. The Frangi filter was utilized with σ_min_ = 0.5, σ_max_ = 5, β = 0.5, *and c* = 15. FasterKAN was configured with a denominator *h* = 0.33, a grid number of 8, a maximum grid of 2, and a minimum grid of −2. Binary cross-entropy and Dice loss were used with weighting coefficients *w*_1_ = 1 and *w*_2_ = 1. Training epochs were set to 1,000. All experiments were conducted on a single NVIDIA A10 GPU.

### 4.2 Evaluation methods

The performance metrics included the Dice similarity coefficient (DSC) (46), sensitivity (Sen) (47), 95% Hausdorff distance (HD95) (48), and Intersection over Union (IoU) (49).

The DSC and sensitivity are defined by Formulas 11, 12:


(11) $$\begin{array}{l} DSC=\frac{2TP}{2TP+FP+FN}\; \end{array}$$

(12) $$\begin{array}{l} Sensitivity=\frac{TP}{TP+FN}\; \end{array}$$

where TP denotes true positive, TN denotes true negative, FP denotes false positive, and FN denotes false negative.

The HD95 means the 95th percentile of the maximum surface distances between segmentation boundaries, evaluating contour alignment precision. This is defined by Formula 13:


(13) $$\begin{array}{l} \begin{array}{c} HD\left(X,Y\right)=\max\left\{h\left(X,Y\right),h\left(Y,X\right)\right\}\\ h\left(X,Y\right)=\max_{x\in X}\min_{y\in Y}d\left(x,y\right)\\ h\left(Y,X\right)=\max_{y\in Y}\min_{x\in X}d\left(x,y\right) \end{array}\; \end{array}$$

where *X* and *Y* denote the ground truth and segmented maps, respectively, and *d*(*x, y*) represents the distance between the points *x* and *y*. HD95 is the 95th percentile of the distances between the boundaries of *X* and *Y*.

IoU, used for comparing the similarity between two arbitrary shapes, is defined by Formula 14:


(14) $$\begin{array}{l} IoU=\frac{\left|A\cap B\right|}{\left|A\cup B\right|}\; \end{array}$$

where *A* and *B* denote two arbitrary shapes representing the ground truth and predicted segmentation maps, respectively.

## 5 Results

We evaluated our method against state-of-the-art approaches under identical experimental conditions. The benchmark architectures included the following: CNN-based models [UNet (9), UNet++ (11), Attention U-Net (12)]; transformer-based methods [TransUNet (14), OCT^2^Former (19), MISSFormer (20)]; and SSM-based approaches [VM-UNet (21) with encoder/decoder weights initialized from ImageNet-1K pretrained VMamba-T (22) and H_vmunet (23)]. All models underwent rigorous 5-fold cross-validation, with a fixed 20% independent test set, and non-pretrained components were trained from scratch.

### 5.1 Quantitative performance evaluation

Table 1 summarizes the comparative results on the clinical dataset. VM-CAGSeg demonstrated superior performance across key metrics, achieving 88.15% DSC, 79.19% mIoU, and 13.68 mm HD95. This outperformed CNN-based approaches [e.g., UNet++ (11): 86.99% DSC, 77.29% mIoU, and 27.15 mm HD95], transformer-based approaches [e.g., MISSFormer (20): 87.94% DSC, 78.80% mIoU, and 16.41 mm HD95; OCT^2^Former (19): 86.47% DSC, 76.35% mIoU, and 17.25 mm HD95], and SSM-based approaches [e.g., H_vmunet (23): 87.24% DSC, 77.76% mIoU, and 18.21 mm HD95]. The method achieved precise boundary delineation with a significantly lower HD95 (13.68 mm) compared to UNet (27.29 mm) and TransUNet (15.85 mm). Although sensitivity (90.05%) was marginally lower than that of TransUNet (90.33%, <0.3%), VM-CAGSeg maintained a superior metric balance, demonstrating robust performance. In summary, VM-CAGSeg excelled in segmentation accuracy (mIoU/DSC) and boundary precision (HD95), making it ideal for clinical applications that require fine-grained segmentation.

**Table 1:** Summary of the comparative results.

**Model**	**mIoU (%)↑**	**DSC (%)↑**	**Sen (%)↑**	**HD95 (mm) ↓**
UNet	77.27	86.97	89.20	27.29
UNet++	77.29	86.99	90.26	27.15
Attention U-Net	76.21	86.02	89.70	30.49
TransUNet	76.88	86.83	**90.33**	15.85
OCT^2^Former	76.35	86.47	88.93	17.25
MISSFormer	78.80	87.94	89.68	16.41
VM-UNet	77.30	87.01	89.50	18.29
H_vmunet	77.76	87.24	89.65	18.21
**VM-CAGSeg**	**79.19**	**88.15**	90.05	**13.68**

The bold value represents the optimal value for this column.

### 5.2 Qualitative performance evaluation

**Figure 5:**
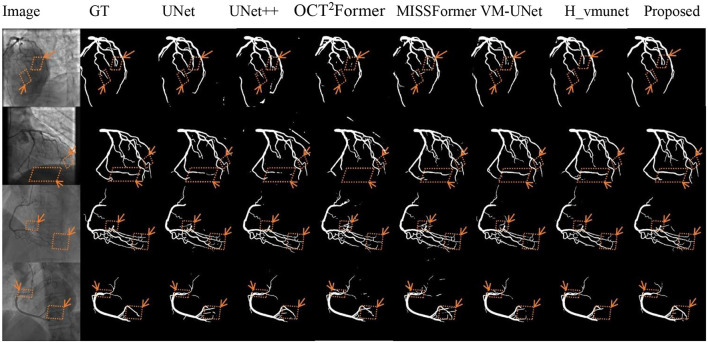
Qualitative evaluation of low-contrast cases. GT denotes the ground truth.

**Figure 6:**
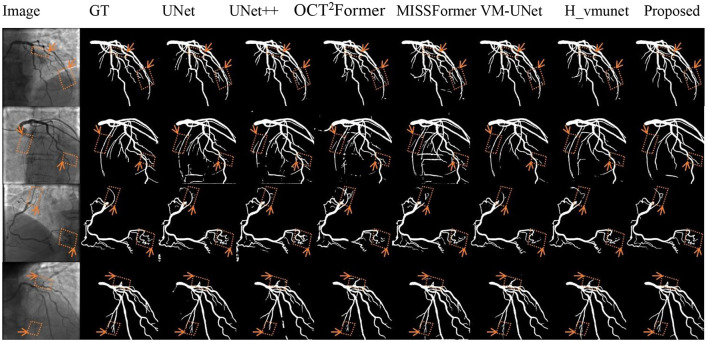
Qualitative evaluation of complex anatomy cases. GT denotes the ground truth.

**Figure 7:**
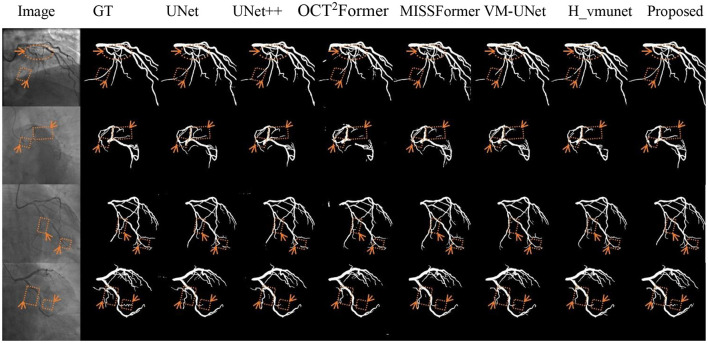
Qualitative evaluation of fuzzy vascular boundary cases. GT denotes the ground truth.

Figures 5–7 present qualitative comparisons between VM-CAGSeg and six state-of-the-art methods (UNet, UNet++, OCT^2^Former, MISSFormer, VM-UNet, H_vmunet) under clinically challenging scenarios. All visualizations used identical preprocessing and displayed parameters to ensure comparability.

Figure 5 shows segmentation performance under low-contrast conditions. VM-CAGSeg preserved complete vascular structures with superior contrast robustness. While UNet and UNet++ exhibited fragmentation in faint branches, the transformer-based methods (OCT^2^Former, MISSFormer) produced discontinuous outputs. The SSM-based approaches (VM-UNet, H_vmunet) maintained better topology but overlooked subtle low-contrast features that were captured by VM-CAGSeg.

Figure 6 shows the segmentation results for complex anatomies. VM-CAGSeg accurately resolved crossing vessels and thin bifurcations without topological errors. The CNN-based methods (UNet/UNet++) produced erroneous inter-vessel connections, while the transformer-based methods (OCT^2^Former, MISSFormer) generated over-segmentation in dense vascular regions. Although the SSM-based methods (VM-UNet, H_vmunet) improved structural accuracy compared to the CNN-based methods, they missed fine branch details that VM-CAGSeg preserved with anatomically precise geometries.

Figure 7 shows segmentation performance at fuzzy vascular boundaries. VM-CAGSeg achieved clinically plausible contours with smooth transitions, particularly in regions with gradual intensity changes. Traditional UNet architectures produced jagged boundaries, while UNet++ showed moderately improved but inconsistent edge smoothness. SSM-based and transformer-based approaches maintained better coherence than CNN-based approaches but lacked VM-CAGSeg's physiological continuity in ambiguous regions.

### 5.3 Ablation studies

In this section, we adopted VM-UNet (22) as the baseline model. We configured a batch size of 4 and used the AdamW optimizer (50) with an initial learning rate of 1e-3. The CosineAnnealingLR scheduler (51) was implemented with a maximum of 50 iterations and a minimum learning rate of 1e-5. Training proceeded for 1,000 epochs. Both encoder and decoder weights were initialized using the Image Net-1k pretrained weights from VMamba-S (21). All the experiments were executed on a single NVIDIA A10 GPU.

#### 5.3.1 Impact of key modules

**Figure 8:**
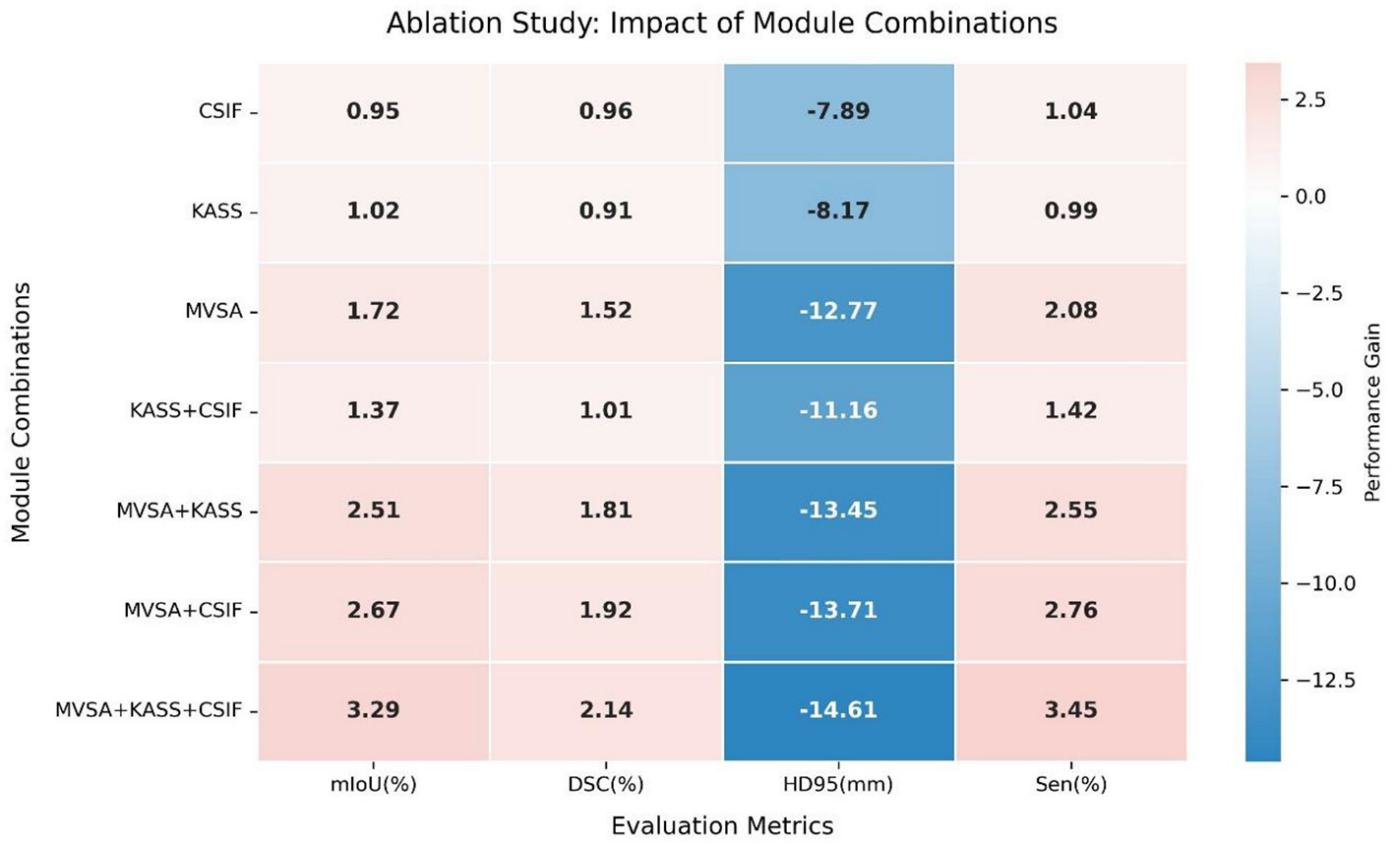
Heatmap of the ablation study.

We conducted ablation experiments on the clinical dataset to assess the contributions of key components. Table 2 shows the incremental improvements from component integration. The baseline model (VM-UNet) achieved a mIoU of 76.30%, DSC of 86.01%, sensitivity (Sen) of 86.50%, and HD95 of 28.29 mm. Adding the CSIF module alone improved mIoU by 0.95%, DSC by 0.96%, and Sen by 1.04% and reduced HD95 by 7.98 mm, highlighting CSIF's ability to enhance cross-stage feature integration. Adding the KASS module alone improved mIoU by 1.02%, DSC by 0.91%, and Sen by 0.99% and reduced HD95 by 8.17 mm. Adding the MVSA module alone improved mIoU by 1.72%, DSC by 1.52%, and Sen by 2.08% and significantly reduced HD95 by 11.77 mm (15.52 mm), validating its ability to capture hierarchical features. Combining the KASS module with the CSIF module yielded 77.67% mIoU, 87.02% DSC, and 87.92% Sen while reducing HD95 to 17.13 mm. Combining the MVSA module with the KASS module further refined performance, increasing mIoU to 78.81%, DSC to 87.82%, and sensitivity (Sen) to 89.05% and reducing HD95 to 14.84 mm. Combining MVSA with CSIF yielded 78.97% mIoU, 87.93% DSC, and 89.26% Sen, while reducing HD95 to 14.58 mm. The full model (MVSA + KASS + CSIF) achieved the best results: 79.59% mIoU, 88.15% DSC, and 13.68 mm HD95. MVSA, KASS, and Cross-Stage Feature Interactive Fusion (CSIF) collectively enhanced segmentation by capturing multiscale vessel structures, modeling complex topological relationships, and fusing cross-stage contexts. The heatmap (Figure 8) conclusively demonstrates that the “MVSA+KASS+CSIF” configuration achieved optimal performance: it yielded the highest values for mIoU, DSC, and Sen while attaining the lowest HD95 metric, confirming that the simultaneous integration of all three modules delivered superior segmentation efficacy.

**Table 2:** Ablation study on the different components of VM-CAGSeg.

**Components**			**mIoU (%)↑**	**DSC (%)↑**	**HD95 (mm)↓**	**Sen (%)↑**
**MVSA**	**KASS**	**CSIF**				
No	No	No	76.30	86.01	28.29	86.50
No	No	Yes	77.25	86.97	20.31	87.54
No	Yes	No	77.32	86.92	20.12	87.49
Yes	No	No	78.02	87.53	15.52	88.58
No	Yes	Yes	77.67	87.02	17.13	87.92
Yes	Yes	No	78.81	87.82	14.84	89.05
Yes	No	Yes	78.97	87.93	14.58	89.26
Yes	Yes	Yes	**79.59**	**88.15**	**13.68**	**89.95**

The bold value represents the optimal value for this column.

#### 5.3.2 Feed-forward network (FFN) comparison

A comparative ablation study evaluated the efficacy of two FFN implementations: multilayer perceptron (MLP) and FasterKAN (32). Table 3 shows the efficacy of two FFN implementations. While the baseline model (VM-UNet) exhibited low computational costs, its performance remained suboptimal (75.30% mIoU, 85.01% DSC, and 28.29 mm HD95). Incorporating a Feed-Forward Network (FFN) into the vanilla VSS block considerably enhanced performance. Adding an MLP to the vanilla VSS block slightly improved accuracy (+0.99% mIoU) but nearly doubled the number of parameters (47.07 M) and FLOPS (8.65 G), making it inefficient. Replacing the MLP with the FasterKAN variant offered a better trade-off, improving mIoU by 2.52% (77.82%) while adding only 8.35 M parameters and minimal FLOPS (4.13 G), validating its efficiency in feature transformation. Therefore, VM-CAGSeg incorporates FasterKAN into the vanilla VSS block.

**Table 3:** Ablation study on the different feed-forward network implementations in VM-CAGSeg.

**Model**	**Params (M)↓**	**FLOPS (G)↓**	**mIoU(%) ↑**	**DSC (%)↑**	**HD95 (%)↓**	**Sen (%)↑**
The baseline model	**22.03**	**4.11**	75.30	85.01	28.29	85.50
The baseline model + MLP	47.07	8.65	76.29	87.04	23.12	87.08
The baseline model + **FasterKAN**	30.38	4.13	**77.82**	**87.53**	**20.12**	**87.58**

The bold value represents the optimal value for this column.

#### 5.3.3 Module placement strategy

In this study, we constrained the MVSA module exclusively to stage 1, leveraging maximal utilization of high-resolution inputs for global vessel topology extraction. Further synergistic division of labor was achieved with downstream topology-refinement modules (KASS). To validate the efficacy of this placement strategy, we conducted an ablation study comparing MVSA configurations across network stages, with quantitative results summarized in Table 4. Critically, exclusive stage 1 deployment achieved optimal performance: 78.02% mIoU, 87.53% DSC, 88.58% Sen, and 15.52 mm HD95. Notably, extending MVSA to subsequent stages increased parameters (22.55 M) and FLOPS (5.33 G) while degrading segmentation efficacy. Contrary to conventional multi-scale processing paradigms, our findings demonstrated that vessel structure awareness delivers peak effectiveness when restricted to initial feature extraction. This strategy aligns with vascular hierarchy principles: macroscopic structures require holistic analysis, whereas microscopic details benefit from localized processing.

**Table 4:** Ablation study on the module placement strategy of MVSA.

**Module placement strategy of MVSA**	**Params (M)↓**	**FLOPS (G)↓**	**mIoU (%) ↑**	**DSC (%) ↑**	**HD95 (%) ↓**	**Sen (%) ↑**
Stage 1	**22.16**	**4.59**	**78.02**	**87.53**	**15.52**	**88.58**
Stage 1–2	22.29	4.87	77.51	87.12	19.15	87.75
Stage 1–3	22.42	5.05	76.82	86.57	23.34	87.03
All stages	22.55	5.33	77.37	87.01	19.97	87.58

The bold value represents the optimal value for this column.

### 5.4 Evaluation of non-angiography vessels

**Figure 9:**
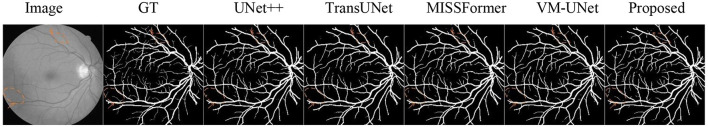
Qualitative evaluation of the DRIVE dataset. GT denotes the ground truth.

We evaluated the proposed network on the benchmark DRIVE (52) retinal vessel dataset. The experimental results demonstrated superior segmentation performance for extremely fine capillaries, visualized in Figure 9 using diamond and circular markers with directional arrows. These findings confirmed the method's robustness and generalization capability.

## 6 Discussion

VM-CAGSeg outperforms existing methods by fundamentally addressing three critical limitations in coronary segmentation: First, geometric-topological synergy. The proposed VSASS block uniquely integrates MVSA's explicit Frangi-based vascular priors with KASS's non-linear state transitions. This combination captures tubular morphology and complex bifurcations more effectively than CNNs (limited receptive fields) or transformers (fixed attention patterns). Second, context-aware fusion. CSFIF overcomes the semantic gap in standard skip connections through cross-stage feature interaction. By dynamically fusing multi-scale contexts, rather than concatenating same-resolution features, it preserves fine-grained details in low-contrast regions where competitors lose vascular continuity. Finally, unified optimization. The architecture co-optimizes structural awareness (MVSA), global dependency modeling (KASS), and hierarchical feature refinement (CSFIF). This holistic approach enables precise boundary delineation in challenging scenarios (e.g., crossing vessels, fuzzy boundaries) where fragmented solutions fail.

VM-CAGSeg effectively addressed three critical challenges in coronary segmentation: low-contrast vessel continuity, complex topological integrity, and boundary ambiguity. Quantitatively, it outperformed CNN-, transformer-, and SSM-based methods across all key metrics, achieving a balance between sensitivity and precision. Qualitatively, it uniquely preserved fine vascular branches in low-contrast regions (Figure 5), resolved bifurcations without errors (Figure 6), and generated physiologically plausible boundaries (Figure 7). While SSM-based approaches showed improved topology, they missed subtle details, and the transformers suffered from over-segmentation. VM-CAGSeg's integration of geometric priors and multi-scale feature refinement enabled unmatched performance in clinically challenging scenarios, making it ideal for fine-grained segmentation tasks.

The superiority of VM-CAGSeg was statistically validated against all compared methods. On the clinical dataset, VM-CAGSeg demonstrated significant improvements in both segmentation accuracy and boundary precision. For DSC, it achieved a mean improvement of +2.5% [95% CI: (1.8%, 3.2%), *p* < 0.001, paired *t*-test] over UNet++ and +1.2% [95% CI: (0.7%,1.7%), *p* = 0.008, Wilcoxon signed-rank test] over MISSFormer. For HD95 (Wilcoxon signed-rank test), it achieved a mean reduction of −13.6 mm [95% CI: (−15.2, 12.0), *p* < 0.001] compared to UNet and −2.9 mm [95% CI: (-3.5,−2.3), *p* =0.002] against VM-UNet. These results confirmed that the observed enhancements were statistically significant and not due to random variability.

## 7 Conclusion and future work

VM-CAGSeg introduces a U-shaped network integrating VSASS blocks and Cross-Stage Feature Interactive Fusion (CSIF) for precise coronary artery segmentation. The architecture incorporates the following: 1) A MVSA module enhancing hierarchical features through Frangi filtering and channel attention, 2) KASS blocks with FasterKAN-accelerated non-linear modeling for topological dependencies, and 3) A CSIF module enabling multi-scale fusion via cross-stage interactions. Evaluated on 1,856 clinical sequences, VM-CAGSeg achieved state-of-the-art performance (88.15% DSC, 79.19% mIoU, and 13.68 mm HD95), with the ablation studies confirming a 3.29% mIoU gain over the baseline. Qualitative validation demonstrates robustness in low-contrast, complex anatomical, and boundary-ambiguous scenarios.

Despite its advantages, there are some limitations: (1) Reliance on Frangi filtering may limit generalization to non-vascular segmentation tasks and (2) computational requirements (30.38 M parameters) could challenge real-time deployment. Future research will focus on the following: (1) Developing lightweight architectures via distillation or quantization, (2) validating generalizability on multi-center datasets, including retinal and cerebral vasculature, (3) extending to 4D spatiotemporal segmentation using angiographic sequences, and (5) exploring weakly-supervised learning to reduce annotation dependence. Integration into interventional planning systems promises to enhance quantitative coronary analysis in clinical practice.

## Data Availability

The raw data supporting the conclusions of this article will be made available by the authors, without undue reservation.
